# Supplementary material for: Molecular mechanisms underlying menthol binding and activation of TRPM8 ion channel
Source: Nat Commun. 2020 Jul 29;11:3790. doi: 10.1038/s41467-020-17582-x (PMC7391767; doi:10.1038/s41467-020-17582-x)
Supplement: Supplementary file 1 — Supplementary Information [file 41467_2020_17582_MOESM1_ESM.pdf]

Molecular mechanisms underlying menthol binding and activation of TRPM8 ion channel

Lizhen Xu[1#], Yalan Han[2,3#], Xiaoying Chen[1], Aerziguli Aierken[1], Han Wen[4], Wenjun Zheng[4], Hongkun Wang[5,6], Xiancui Lu[2,3], Zhenye Zhao[1], Cheng Ma[7], Ping Liang[5,6], Wei Yang[8]*, Shilong Yang[9]* and Fan Yang[1]*

[1]Department of Biophysics, and Kidney Disease Center of the First Affiliated Hospital, Zhejiang University School of Medicine, Hangzhou 310058, Zhejiang Province, China.

[2]Key Laboratory of Animal Models and Human Disease Mechanisms of Chinese Academy of Sciences/Key Laboratory of bioactive peptides of Yunnan Province, Kunming Institute of Zoology, Kunming 650223, Yunnan, China.

[3]University of Chinese Academy of Sciences, Beijing, China

[4]Department of Physics, State University of New York at Buffalo, Buffalo, New York, U.S.A.

[5]Key Laboratory of Combined Multi-organ Transplantation, Ministry of Public Health, The First Affiliated Hospital, Zhejiang University School of Medicine

[6]Institute of Translational Medicine, Zhejiang University

[7]Protein facility, School of Medicine, Zhejiang University

[8]Department of Biophysics, and Department of Neurosurgery of the First Affiliated Hospital, Zhejiang University School of Medicine, Hangzhou 310058, Zhejiang Province, China

[9]College of Wildlife and Protected Area, Northeast Forestry University, Harbin 150040, China

[#]These authors contributed equally: Lizhen Xu and Yalan Han

Correspondence and requests for materials should be addressed to W.Y. (email: yangwei@zju.edu.cn) or to S.-L.Y. (email: syang2020@nefu.edu.cn) or to F.Y. (email: fanyanga@zju.edu.cn)

Supplementary Figures

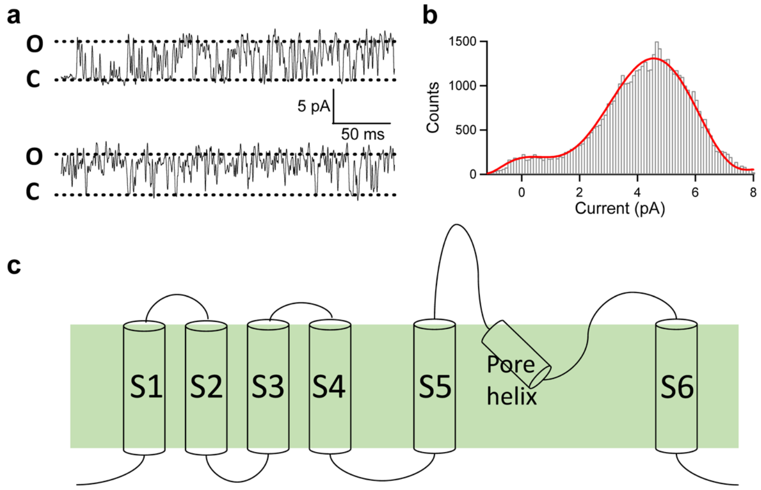

Supplementary Figure 1. Single-channel recording of menthol activation of TRPM8 channel. (**a**) Representative single-channel current traces of TRPM8 channel in response to 500 µM menthol recorded in the inside-out configuration. (**b**) All-point histogram of the representative single-channel traces. The histogram was fitted to a double gauss function (line in red). (**c**) A diagram illustrating the topology of TRPM8 transmembrane domains.

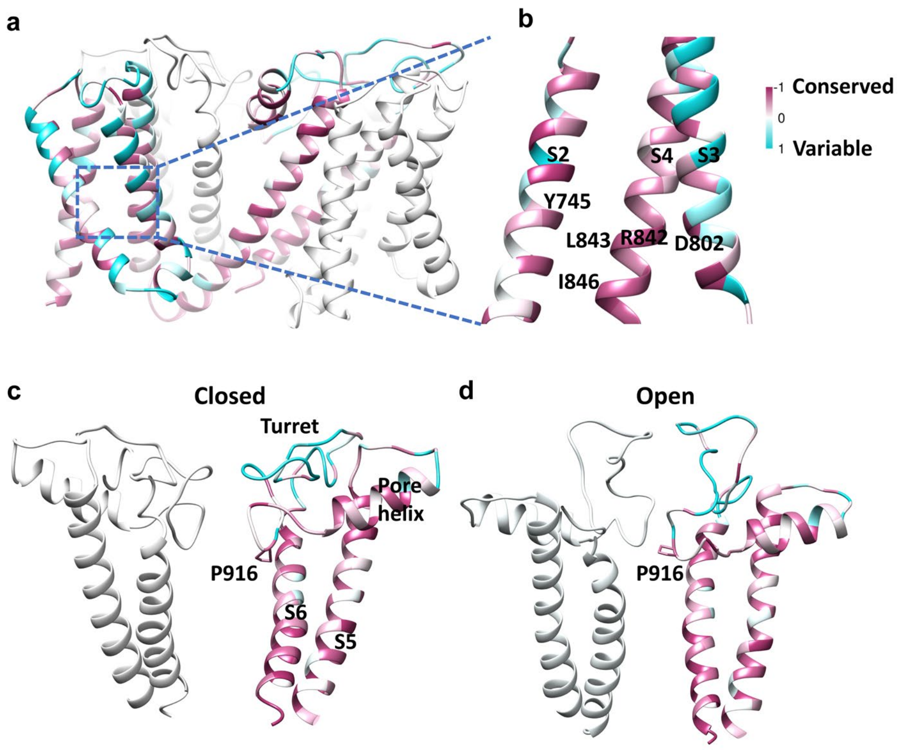

Supplementary Figure 2. ConSurf analysis of TRPM8 sequence conservation. (**a**) One subunit of TRPM8 was colored based on the protein sequence conservation score calculated by the ConSurf server. Residues in maroon and cyan are evolutionarily conserved or variable, respectively. (**b**) Zoom-in view of the ligand binding pocket formed in the S1-S4 domain. Residues highly conserved are also critical for menthol binding. (**c** and **d**) Residue conservation scores were mapped to the pore region in the closed (**c**) and potential open (**d**) state models.

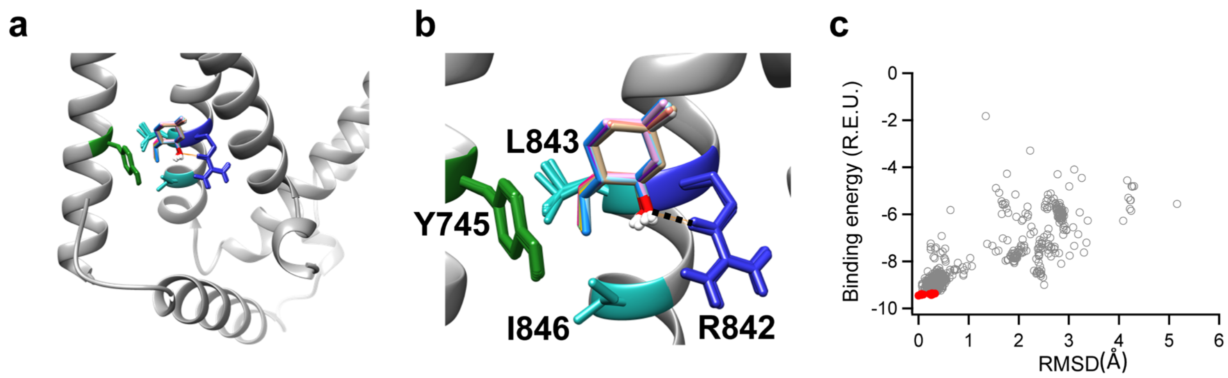

Supplementary Figure 3. Docking of menthol molecule into the potential open state model. (**a**) The top 10 docking model with lowest binding energy. R842, Y745 and L843 and I846 were colored in blue, green and cyan, respectively. (**b**) The zoom in view of the menthol binding configuration. A hydrogen bond is formed between the hydroxyl group of menthol and R842 (dashed line in black). (**c**) The docking models exhibited a funnel-shaped distribution of binding energy calculated by Rosetta (R.E.U., Rosetta energy unit). The top 10 models with lowest binding energy were colored in red.

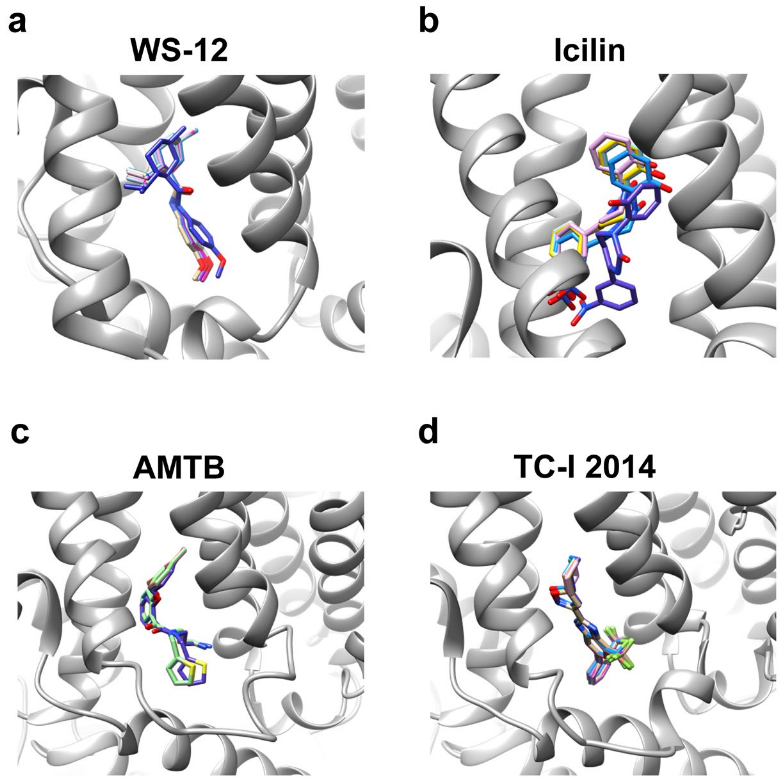

Supplementary Figure 4. Docking of ligands into TRPM8. (**a** to **d**) The top 10 docking model of WS-12, icilin, AMTB and TC-I 2014 with lowest binding energy into the corresponding structural states of TRPM8 (PDB ID: 6NR2, 6NR3, 6O6R and 6O72, respectively).

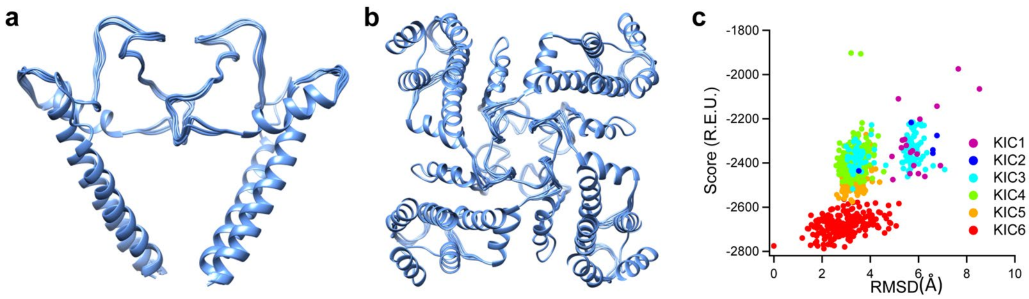

Supplementary Figure 5. Modeling of TRPM8 in the menthol-induced open state. (**a** and **b**) The side (**a**) and button (**b**) view of top 10 models with lowest energy after six rounds of KIC loop modeling. These models were well converged. (**c**) The potential models of the open state exhibited a funnel-shaped distribution of total energy after six rounds of KIC loop modeling as calculated by Rosetta (R.E.U., Rosetta energy unit).

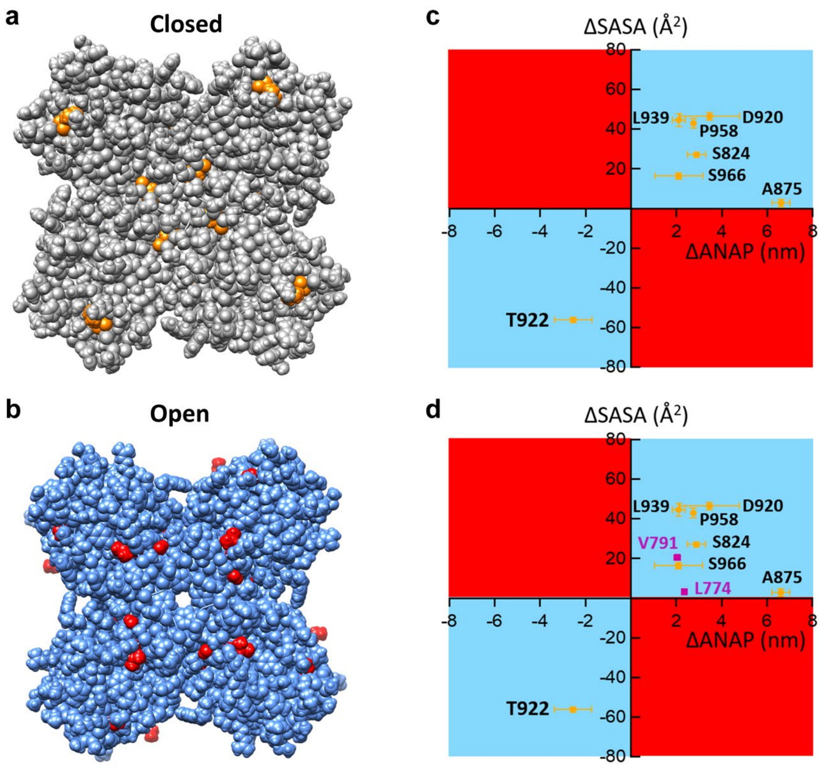

Supplementary Figure 6. Shifts in ANAP emission positively correlated with changes in SASA. (**a** and **b**) The top view of TRPM8 models in the closed (a) and open (b) states. All atoms in residues were showed in a sphere mode. The residues showing a shift in ANAP emission upon menthol activation were labeled in orange in the closed state (**a**) and in red in the open state (**b**), respectively. (**c** and **d**) Positive correlation between shifts in ANAP emission peak (y axis) and changes in SASA measured from our closed and open state models (x axis); n = 4–6. ANAP shifts measured from L774 and V791 were not used in modeling building. Their ANAP shifts were also in agreement with changes in SASA predicted from our models (**d**, data points in purple). All statistical data are given as mean ± s.e.m.

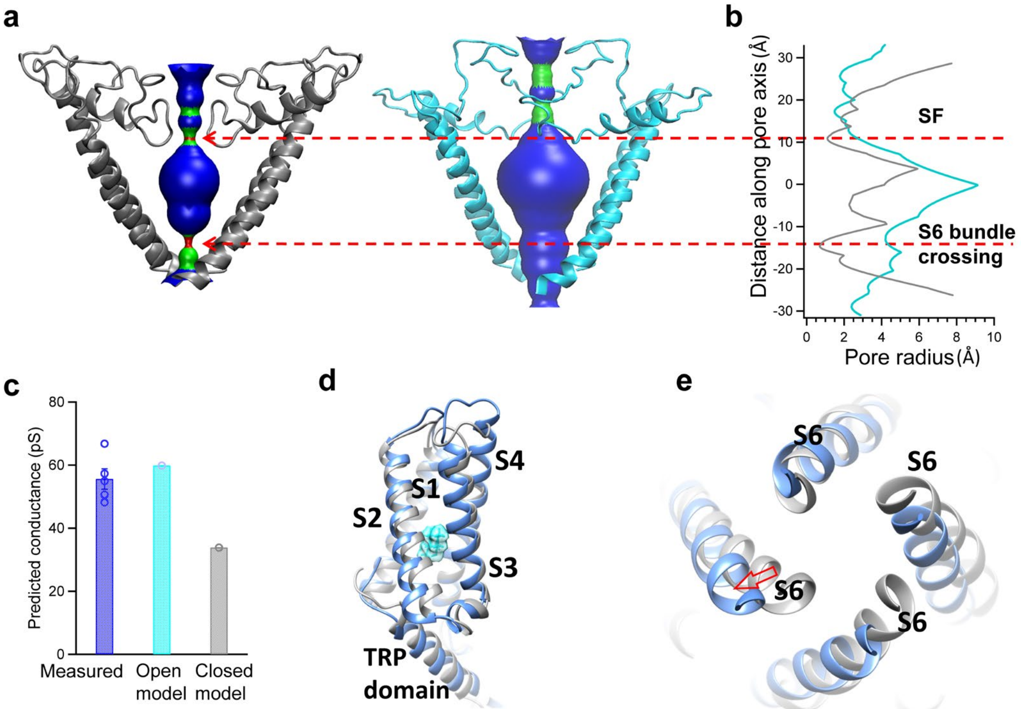

Supplementary Figure 7. **A potential model of menthol-induced open state channel.** (**a** and **b**) Distribution of pore radii in our models of TRPM8 channel in the closed (grey) and potential open (cyan) state, respectively. In our model of the open state, both the selectivity filer region and the S6 bundle crossing are wide enough for water molecules and ions to permeate. (**c**) Conductance of TRPM8 in different states predicted by the HOLE program. Both our experimentally measured conductance (55.5 ± 3.2 pS, n=5; also see in **Fig. S1**) and predicted conductance for the open state model were much larger than the predicted conductance for the closed state model. (**d** and **e**) Menthol-induced conformational rearrangements as suggested by comparing TRPM8 structural models in the closed (grey) and open (cyan) state. All statistical data are given as mean ± s.e.m.

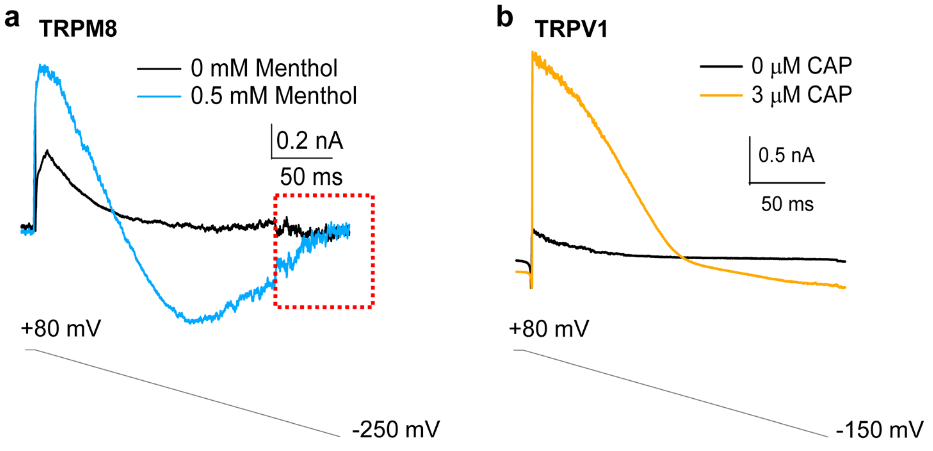

Supplementary Figure 8. Deep hyperpolarization antagonized menthol activation of TRPM8 but not capsaicin activation of TRPV1. (**a**) Representative whole-cell patch-clamp recording of menthol activation of TRPM8 in response to a voltage ramp. In the hyperpolarized voltage range, TRPM8 current increased first and then decreased towards the baseline. Such a hook-shaped current indicated that at deep hyperpolarized voltage the open probability was decreased towards zero even in the presence of 0.5 mM menthol. (**b**) Representative whole-cell patch-clamp recording of capsaicin activation of TRPV1 in response to a voltage ramp. In the hyperpolarized voltage range, TRPV1 current kept increasing linearly with the lowering of membrane potential, which indicated that the channel open probability stayed at a constant level.

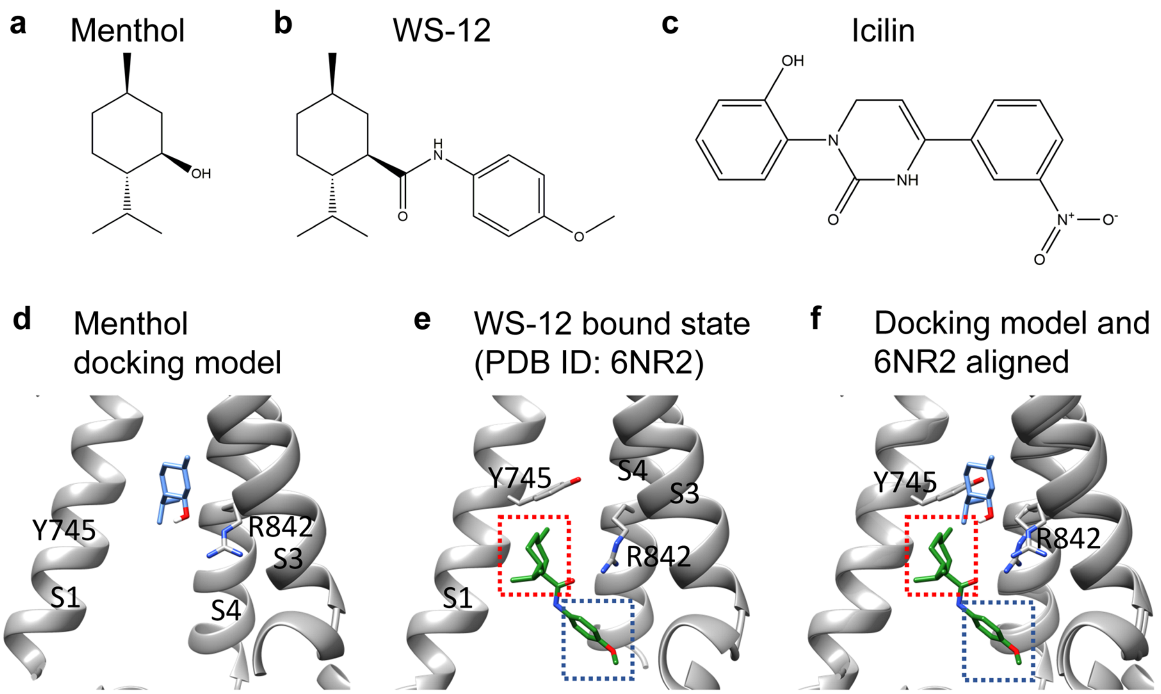

Supplementary Figure 9. Comparison of TRPM8 agonists and their binding configurations. (**a** to **c**) Chemical structures of menthol (**a**), WS-12 (**b**) and icilin (**c**), respectively. (**d**) Our menthol docking model. (**e**) cryo-EM structure of the S1-S4 domain with WS-12 bound. The menthol-like moiety and benzene ring like moiety of WS-12 were highlighted by dashed boxes in red and blue, respectively. (**f**) Comparison of our menthol docking model and WS-12 bound state cryo-EM structure. The menthol-like moiety and benzene ring like moiety of WS-12 were highlighted by dashed boxes in red and blue, respectively.

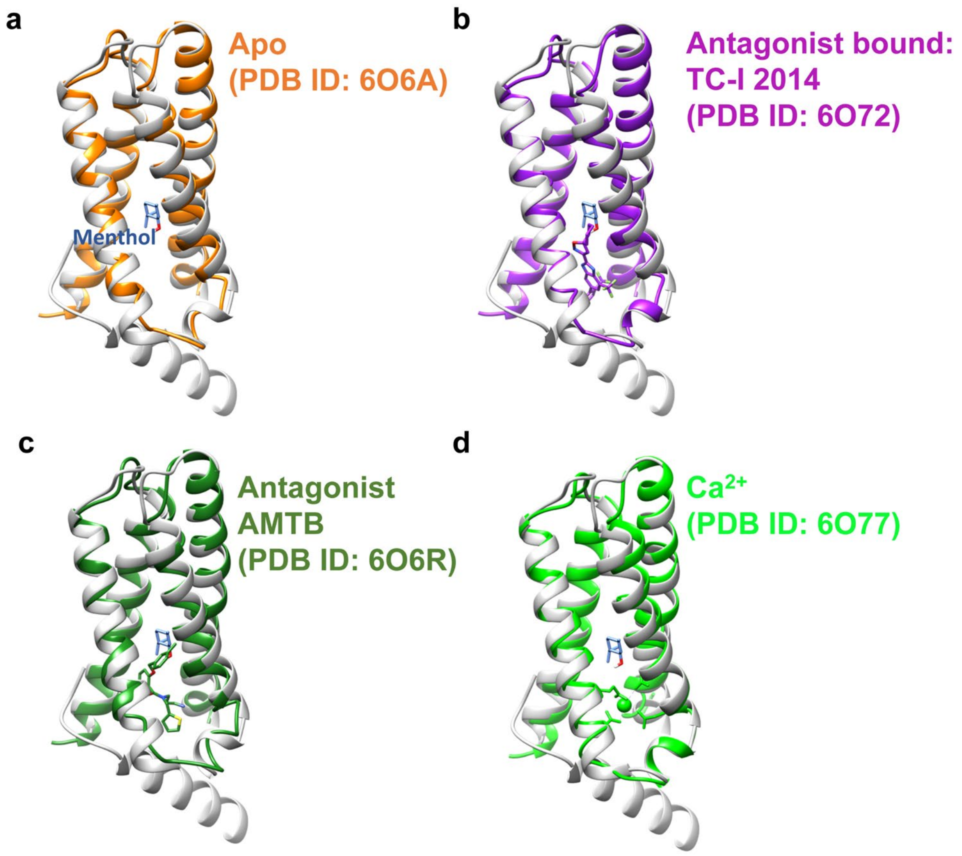

Supplementary Figure 10. Comparison of the S1-S4 domain in menthol docking model with parrot TRPM8 ligand bound states. Menthol docking model was colored in grey.

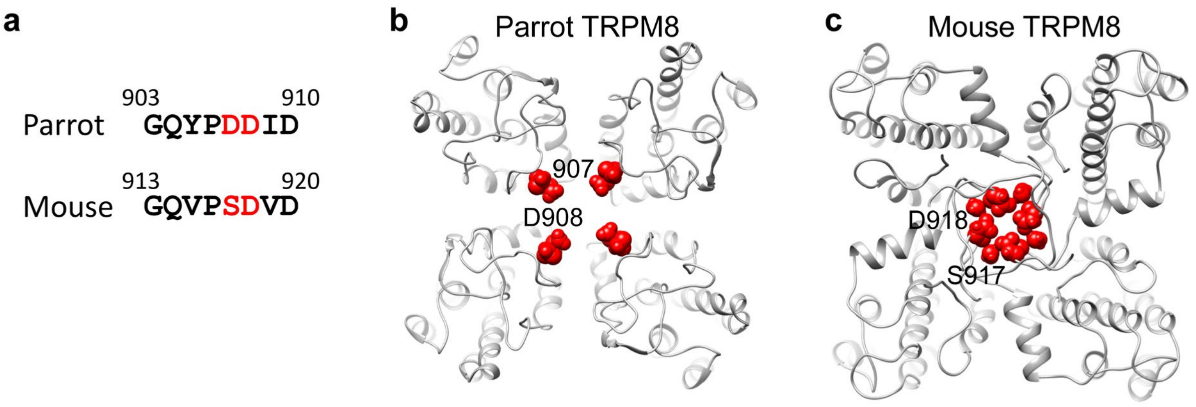

Supplementary Figure 11. Comparison of the selectivity filter in parrot and mouse TRPM8. (**a**) Amino acid sequence alignment of the selectivity filter in TRPM8 channels. (**b** and **c**) Top view of the parrot TRPM8 in the desensitized state (PDB ID: 6O77) and our model of menthol activated state of mouse TRPM8. Residues toward the ion permeation pathway were highlighted in red.

Supplementary Tables

Supplementary Table 1. ANAP-incorporated TRPM8 mutants. The function of these ANAP-incorporated mutants was accessed by patch-clamp recordings. Menthol (1 mM) was perfused to activate a mutant channel.

Mutant	Position	Menthol activation
P734ANAP	S1	YES
N741ANAP	S1	NO
L756ANAP	S1	NO
D759ANAP	S1S2 linker	NO
H761ANAP	S1S2 linker	NO
P764ANAP	S1S2 linker	NO
L774ANAP	S2	YES
Y787ANAP	S2	YES
N789ANAP	S2S3 linker	YES
V791ANAP	S2S3 linker	YES
T795ANAP	S2S3 linker	YES
Y808ANAP	S3	YES
L817ANAP	S3	YES
S819ANAP	S3-S4 linker	YES
S820ANAP	S3-S4 linker	YES
N821ANAP	S3-S4 linker	YES
K822ANAP	S3-S4 linker	NO
S823ANAP	S3-S4 linker	YES
S824ANAP	S4	YES
N852ANAP	S4	YES
L860ANAP	S5	NO
L864ANAP	S5	YES
I865ANAP	S5	NO
F870ANAP	S5	NO
F872ANAP	S5	NO
L873ANAP	S5	NO
F874ANAP	S5	NO
A875ANAP	S5	YES
V876ANAP	S5	YES
W877ANAP	S5	NO
M878ANAP	S5	NO

V879ANAP	S5	NO
A880ANA	S5	NO
F881ANAP	S5	NO
G882ANAP	S5	NO
V883ANAP	S5	NO
A884ANAP	S5	NO
R885ANAP	S5	NO
Q886ANAP	S5	NO
G887ANAP	S5	NO
Q894ANAP	Between S5 and S6	NO
D920ANAP	Between S5 and S6	YES
S921ANAP	Between S5 and S6	YES
S932ANAP	Between S5 and S6	NO
L939ANAP	Between S5 and S6	YES
D944ANAP	Between S5 and S6	NO
N947ANAP	Between S5 and S6	YES
P952ANAP	Between S5 and S6	YES
W954ANAP	Between S5 and S6	YES
I955ANAP	Between S5 and S6	NO
T956ANAP	Between S5 and S6	NO
I957ANAP	Between S5 and S6	NO
P958ANAP	Between S5 and S6	YES
L959ANAP	S6	NO
V960ANAP	S6	NO
C961ANAP	S6	NO
I962ANAP	S6	YES
Y963ANAP	S6	NO
M964ANAP	S6	NO
L965ANAP	S6	NO
S966ANAP	S6	YES
T967ANAP	S6	NO
N968ANAP	S6	NO
I969ANAP	S6	NO
Q992ANAP	TRP domain	NO
W994ANAP	TRP domain	NO
K995ANAP	TRP domain	NO

Q997ANAP	TRP domain	NO
R998ANAP	TRP domain	NO
Y999ANAP	TRP domain	YES
Q1003ANAP	TRP domain	YES
E1004ANAP	TRP domain	NO
C1006ANAP	TRP domain	YES

Supplementary Table 2. Primers used in this study to generate point mutations.

Name	Primer-Forward	Primer-Reverse
Y745F	CGTGGTCTTCTTCATCGCCTTCCTCCTGCTGTTTGCCT	GGAAGGCGATGAAGAAGACCACGTTCCAGGAGAAGACC
Y745H	CGTGGTCTTCCACATCGCCTTCCTCCTGCTGTTTGCCT	GGAAGGCGATGTGGAAGACCACGTTCCAGGAGAAGACC
Y745W	CGTGGTCTTCTGGATCGCCTTCCTCCTGCTGTTTGCCT	GGAAGGCGATCCAGAAGACCACGTTCCAGGAGAAGACC
D802L	GAACGTTATGTTAACCCTGGGACTCTTCTACTTCATAG	GTCCCAGGGTTAACATAACGTTCCATAGGTCGGTGAAA
D802S	GAACGTTATGTCCACCCTGGGACTCTTCTACTTCATAG	GTCCCAGGGTGGACATAACGTTCCATAGGTCGGTGAAA
D802I	GAACGTTATGATCACCCTGGGACTCTTCTACTTCATAG	GTCCCAGGGTGATCATAACGTTCCATAGGTCGGTGAAA
Y1005F	GGTGCAGGAGGCCTGCAACCGCCTAAACATCCCCTTCC	GGCGGTTGCAGGCCTCCTGCACCAGGAAGTACCGCTGG
R842K	ATTCACGCTAAAGCTCATCCACATTTTCACCGTCAGCA	TGTGGATGAGCTTTAGCGTGAATATAATGTAATCCAGA
L843A	CACGCTAAGGGCCATCCACATTTTCACCGTCAGCAGGA	AAATGTGGATGGCCCTTAGCGTGAATATAATGTAATCC
V775A	CTACGCCCTGGCCTTCGTCCTCTTCTGTGATGAAGTGA	AGAGGACGAAGGCCAGGGCGTAGAGGATCAGCTCGGGG
L843I	CACGCTAAGGATCATCCACATTTTCACCGTCAGCAGGA	AAATGTGGATGATCCTTAGCGTGAATATAATGTAATCC
L843V	CACGCTAAGGGTCATCCACATTTTCACCGTCAGCAGGA	AAATGTGGATGACCCTTAGCGTGAATATAATGTAATCC
L846V	GCTCATCCACGTTTTCACCGTCAGCAGGAACTTGGGAC	TGACGGTGAAAACGTGGATGAGCCTTAGCGTGAATATA
F839A	TTACATTATAGCCACGCTAAGGCTCATCCACATTTTCA	GCCTTAGCGTGGCTATAATGTAATCCAGACAGAAAATG
L797A	TTTCACCGACGCATGGAACGTTATGGACACCCTGGGAC	TAACGTTCCATGCGTCGGTGAAATAATTCACTCCGTTC
W798A	CACCGACCTAGCGAACGTTATGGACACCCTGGGACTCT	CCATAACGTTCGCTAGGTCGGTGAAATAATTCACTCCG
S819ANAP	TCGGTTGCACTAGAGCGACGAGTCCTCCTGGTACAGCG	ACTCGTCGCTCTAGTGCAACCGAAACACGATGCCGGCG
S820ANAP	GTTGCACTCCTAGGACGAGTCCTCCTGGTACAGCGGGA	AGGACTCGTCCTAGGAGTGCAACCGAAACACGATGCCG
D821ANAP	GCACTCCAGCTAGGAGTCCTCCTGGTACAGCGGGAGGG	AGGAGGACTCCTAGCTGGAGTGCAACCGAAACACGATG
E822ANAP	CTCCAGCGACTAGTCCTCCTGGTACAGCGGGAGGGTCA	ACCAGGAGGACTAGTCGCTGGAGTGCAACCGAAACACG

S823ANAP	CAGCGACGAGTAGTCCTGGTACAGCGGGAGGGTCATTT	TGTACCAGGACTACTCGTCGCTGGAGTGCAACCGAAAC
S824ANAP	CGACGAGTCCTAGTGGTACAGCGGGAGGGTCATTTTCT	CGCTGTACCACTAGGACTCGTCGCTGGAGTGCAACCGA
N852ANAP	AGTGTCTAGGTAGCTCGGGCCTAAGATTATCATGCTCC	TAGGCCCGAGCTACCTAGACACTGTGAAGATGTGAATC
L860ANAP	GATTATCATGTAGCAGAGGATGATGATTGACGTCTTCT	TCATCCTCTGCTACATGATAATCTTAGGCCCGAGGTTC
M864ANAP	CCAGAGGATGTAGATTGACGTCTTCTTCTTTTTGTTTT	AGACGTCAATCTACATCCTCTGGAGCATGATAATCTTA
I865ANAP	GAGGATGATGTAGGACGTCTTCTTCTTTTTGTTTTTGT	AGAAGACGTCCTACATCATCCTCTGGAGCATGATAATC
F870ANAP	CGTCTTCTTCTAGTTGTTTTTGTTCGCCGTGTGGATGG	ACAAAAACAACTAGAAGAAGACGTCAATCATCATCCTC
F872ANAP	CTTCTTTTTGTAGTTGTTCGCCGTGTGGATGGTGGCCT	CGGCGAACAACTACAAAAAGAAGAAGACGTCAATCATC
L873ANAP	CTTTTTGTTTTAGTTCGCCGTGTGGATGGTGGCCTTTG	ACACGGCGAACTAAAACAAAAAGAAGAAGACGTCAATC
F874ANAP	TTTGTTTTTGTAGGCCGTGTGGATGGTGGCCTTTGGGG	TCCACACGGCCTACAAAAACAAAAAGAAGAAGACGTCA
A875ANAP	GTTTTTGTTCTAGGTGTGGATGGTGGCCTTTGGGGTGG	CCATCCACACCTAGAACAAAAACAAAAAGAAGAAGACG
V876ANAP	TTTGTTCGCCTAGTGGATGGTGGCCTTTGGGGTGGCCC	CCACCATCCACTAGGCGAACAAAAACAAAAAGAAGAAG
W877ANAP	GTTCGCCGTGTAGATGGTGGCCTTTGGGGTGGCCCGGC	AGGCCACCATCTACACGGCGAACAAAAACAAAAAGAAG
M878ANAP	CGCCGTGTGGTAGGTGGCCTTTGGGGTGGCCCGGCAGG	CAAAGGCCACCTACCACACGGCGAACAAAAACAAAAAG
V879ANAP	CGTGTGGATGTAGGCCTTTGGGGTGGCCCGGCAGGGCA	TGCCCTGCCGGGCCACCCCAAAGGCCTACATCCACACG
A880ANAP	GTGGATGGTGTAGTTTGGGGTGGCCCGGCAGGGCATCC	CCACCCCAAACTACACCATCCACACGGCGAACAAAAAC
F881ANAP	GATGGTGGCCTAGGGGGTGGCCCGGCAGGGCATCCTGA	GGGCCACCCCCTAGGCCACCATCCACACGGCGAACAAA
G882ANAP	GGTGGCCTTTTAGGTGGCCCGGCAGGGCATCCTGAGGA	GCCGGGCCACCTAAAAGGCCACCATCCACACGGCGAAC
V883ANAP	GGCCTTTGGGTAGGCCCGGCAGGGCATCCTGAGGAAGA	CCTGCCGGGCCTACCCAAAGGCCACCATCCACACGGCG
A884ANAP	CTTTGGGGTGTAGCGGCAGGGCATCCTGAGGAAGAACG	TGCCCTGCCGCTACACCCCAAAGGCCACCATCCACACG
R885ANAP	TGGGGTGGCCTAGCAGGGCATCCTGAGGAAGAACGAGC	GGATGCCCTGCTAGGCCACCCCAAAGGCCACCATCCAC

Q886ANAP	GGTGGCCCGGTAGGGCATCCTGAGGAAGAACGAGCATA	TCAGGATGCCCTACCGGGCCACCCCAAAGGCCACCATC
G887ANAP	GGCCCGGCAGTAGATCCTGAGGAAGAACGAGCATAGAT	TCCTCAGGATCTACTGCCGGGCCACCCCAAAGGCCACC
H894ANAP	GAAGAACGAGTAGAGATGGGAGTGGATTTTTAGATCCG	ACTCCCATCTCTACTCGTTCTTCCTCAGGATGCCCTGC
D920ANAP	AGATGATGTGTAGGGGACAACATATAACTTTGATAGGT	ATGTTGTCCCCTACACATCATCTGGGTACTGGCCGAAC
G921ANAP	TGATGTGGATTAGACAACATATAACTTTGATAGGTGTA	TATATGTTGTCTAATCCACATCATCTGGGTACTGGCCG
S932ANAP	GTGTACCTTTTAGGGCAATGAGTCCAAGCCCCTGTGCG	ACTCATTGCCCTAAAAGGTACACCTATCAAAGTTATAT
L939ANAP	GTCCAAGCCCTAGTGCGTGGAGCTGGACGCCAACAACC	GCTCCACGCACTAGGGCTTGGACTCATTGCCGCTAAAG
D944ANAP	CGTGGAGCTGTAGGCCAACAACCAGCCTCGGTTTCCCG	GGTTGTTGGCCTACAGCTCCACGCACAGGGGCTTGGAC
N947ANAP	GGACGCCAACAACCAGCCTCGGTTTCCCGAGTGGATCA	ACCGAGGCTGCTAGTTGGCGTCCAGCTCCACGCACAGG
P952ANAP	GCCTCGGTTTTAGGAGTGGATCACCATCCCCCTGGTGT	TGATCCACTCCTAAAACCGAGGCTGGTTGTTGGCGTCC
W954ANAP	GTTTCCCGAGTAGATCACCATCCCCCTGGTGTGCATCT	GGATGGTGATCTACTCGGGAAACCGAGGCTGGTTGTTG
I955ANAP	TCCCGAGTGGTAGACCATCCCCCTGGTGTGCATCTACA	GGGGGATGGTCTACCACTCGGGAAACCGAGGCTGGTTG
T956ANAP	CGAGTGGATCTAGATCCCCCTGGTGTGCATCTACATGC	CCAGGGGGATCTAGATCCACTCGGGAAACCGAGGCTGG
I957ANAP	GTGGATCACCTAGCCCCTGGTGTGCATCTACATGCTGA	ACACCAGGGGCTAGGTGATCCACTCGGGAAACCGAGGC
P958ANAP	GATCACCATCTAGCTGGTGTGCATCTACATGCTGAGCA	TGCACACCAGCTAGATGGTGATCCACTCGGGAAACCGA
L959ANAP	CACCATCCCCTAGGTGTGCATCTACATGCTGAGCACAA	AGATGCACACCTAGGGGATGGTGATCCACTCGGGAAAC
V960ANAP	CATCCCCCTGTAGTGCATCTACATGCTGAGCACAAACA	TGTAGATGCACTACAGGGGGATGGTGATCCACTCGGGA
C961ANAP	CCCCCTGGTGTAGATCTACATGCTGAGCACAAACATTT	GCATGTAGATCTACACCAGGGGGATGGTGATCCACTCG
I962ANAP	CCTGGTGTGCTAGTACATGCTGAGCACAAACATTTTGC	TCAGCATGTACTAGCACACCAGGGGGATGGTGATCCAC
Y963ANAP	GGTGTGCATCTAGATGCTGAGCACAAACATTTTGCTCG	TGCTCAGCATCTAGATGCACACCAGGGGGATGGTGATC
M964ANAP	GTGCATCTACTAGCTGAGCACAAACATTTTGCTCGTGA	TTGTGCTCAGCTAGTAGATGCACACCAGGGGGATGGTG

L965ANAP	CATCTACATGTAGAGCACAAACATTTTGCTCGTGAACT	TGTTTGTGCTCTACATGTAGATGCACACCAGGGGGATG
S966ANAP	CTACATGCTGTAGACAAACATTTTGCTCGTGAACTTGC	AAATGTTTGTCTACAGCATGTAGATGCACACCAGGGGG
T967ANAP	CATGCTGAGCTAGAACATTTTGCTCGTGAACTTGCTGG	GCAAAATGTTCTAGCTCAGCATGTAGATGCACACCAGG
N968ANAP	GCTGAGCACATAGATTTTGCTCGTGAACTTGCTGGTGG	CGAGCAAAATCTATGTGCTCAGCATGTAGATGCACACC
I969ANAP	GAGCACAAACTAGTTGCTCGTGAACTTGCTGGTGGCCA	TCACGAGCAACTAGTTTGTGCTCAGCATGTAGATGCAC
Q992ANAP	GAACAACGATTAGGTGTGGAAGTTCCAGCGGTACTTCC	ACTTCCACACCTAATCGTTGTTCTCCTGGACGCTGCCG
W994ANAP	CGATCAGGTGTAGAAGTTCCAGCGGTACTTCCTGGTGC	GCTGGAACTTCTACACCTGATCGTTGTTCTCCTGGACG
K995ANAP	TCAGGTGTGGTAGTTCCAGCGGTACTTCCTGGTGCAGG	ACCGCTGGAACTACCACACCTGATCGTTGTTCTCCTGG
Q997ANAP	GTGGAAGTTCTAGCGGTACTTCCTGGTGCAGGAGTACT	GGAAGTACCGCTAGAACTTCCACACCTGATCGTTGTTC
R998ANAP	GAAGTTCCAGTAGTACTTCCTGGTGCAGGAGTACTGCA	CCAGGAAGTACTACTGGAACTTCCACACCTGATCGTTG
Y999ANAP	GTTCCAGCGGTAGTTCCTGGTGCAGGAGTACTGCAGCA	GCACCAGGAACTACCGCTGGAACTTCCACACCTGATCG
Q1003ANAP	CTTCCTGGTGTAGGAGTACTGCAGCAGGCTGACCATCC	TGCAGTACTCCTACACCAGGAAGTACCGCTGGAACTTC
E1004ANAP	CCTGGTGCAGTAGTACTGCAGCAGGCTGACCATCCCCT	TGCTGCAGTACTACTGCACCAGGAAGTACCGCTGGAAC
C1006ANAP	GCAGGAGTACTAGAGCAGGCTGACCATCCCCTTTCCAT	TCAGCCTGCTCTAGTACTCCTGCACCAGGAAGTACCGC

Supplementary Methods

Commands in Rosetta to perform loop modeling:

```
/home/fanyang/rosetta/main/source/bin/loopmodel.linuxgccrelease \
-in:path:database /home/fanyang/rosetta2015.25/main/database \
-score:weights membrane_highres_Menv_smooth.wts \
-in:file:fullatom \
-membrane:normal_cycles 100 \
-membrane:normal_mag 15 \
-membrane:center_search \
-ignore_unrecognized_res \
-symmetry:symmetry_definition
/home/fanyang/projects/input_files/3J5R_2017/4D_ABCD_r_4D_after_ccd_relaxed_r.symm \
-symmetry:initialize_rigid_body_dofs \
-in:file:spanfile /###.span \
-in:file:s /###.pdb \
-loops:loop_file /###.loop \
-loops:remodel perturb_kic \
-loops:refine refine_kic \
-loops:relax no \
-loops:strict_loops \
-loops:build_attempts 20 \
-relax:bb_move false \
-max_inner_cycles 30 \
-nstruct 51 \
-out:prefix msymm-loop-kic- \
-out:file:silent /###.silent \
```

```
-out:file:silent_struct_type binary \
-mute all
```

Commands in Rosetta to perform SASA calculation and filtering:

```
/home/fanyang/rosetta/main/source/bin/rosetta_scripts.linuxgccrelease \
-database /home/fan/rosetta_2016.20/main/database
-membrane:normal_cycles 100
-membrane:normal_mag 15
-membrane:center_search
-in:file:spanfile /###.span
-score:weights membrane_highres_Menv_smooth.wts
-parser:protocol /###.xml
-symmetry:symmetry_definition /###.symm
-symmetry:initialize_rigid_body_dofs
-ignore_unrecognized_res
-in:file:silent_struct_type binary
-in:file:silent /###.out
-nstruct 1
-out:file:silent /###.silent
-out:file:silent_struct_type binary
-overwrite
```

Rosetta scripts to perform SASA calculation and filtering:

```
<ROSETTASCRIPTS>
    <RESIDUE_SELECTORS>
      <Not name="A875_unselected">
           <Index resnums=143/>
      </Not>
      <Not name="D920_unselected">
           <Index resnums=188/>
      </Not>
        <Not name="L939_unselected">
                <Index resnums=207/>
        </Not>
        <Not name="P958_unselected">
                <Index resnums=226/>
        </Not>
        <Not name="S966_unselected">
                <Index resnums=234/>
        </Not>
    </RESIDUE_SELECTORS>
    <TASKOPERATIONS>
      <OperateOnResidueSubset name="A875_only"
selector="A875_unselected" >
           <PreventRepackingRLT/>
      </OperateOnResidueSubset>
      <OperateOnResidueSubset name="D920_only"
selector="D920_unselected" >
           <PreventRepackingRLT/>
      </OperateOnResidueSubset>
        <OperateOnResidueSubset name="L939_only"
selector="L939_unselected" >
           <PreventRepackingRLT/>
        </OperateOnResidueSubset>
      <OperateOnResidueSubset name="P958_only"
selector="P958_unselected" >
           <PreventRepackingRLT/>
      </OperateOnResidueSubset>
      <OperateOnResidueSubset name="S966_only"
selector="S966_unselected" >
           <PreventRepackingRLT/>
      </OperateOnResidueSubset>
    </TASKOPERATIONS>
    <SCOREFXNS>
    </SCOREFXNS>
 <FILTERS>
        <TotalSasa name=D920_sasa threshold=10
task_operations=D920_only report_per_residue_sasa=1/>
        <TotalSasa name=L939_sasa threshold=10
task_operations=L939_only report_per_residue_sasa=1/>
        <TotalSasa name=P958_sasa threshold=10
task_operations=P958_only report_per_residue_sasa=1/>
```

```
        <TotalSasa name=S966_sasa threshold=30
task_operations=S966_only report_per_residue_sasa=1/>
        <TotalSasa name=A875_sasa threshold=48
task_operations=A875_only report_per_residue_sasa=1/>
 </FILTERS>
    <MOVERS>
    </MOVERS>
    <PROTOCOLS>
        <Add filter=D920_sasa/>
        <Add filter=L939_sasa/>
        <Add filter=P958_sasa/>
        <Add filter=S966_sasa/>
        <Add filter=A875_sasa/>
    </PROTOCOLS>

</ROSETTASCRIPTS>
```
