# Supplementary material for: Molecular mechanisms underlying menthol binding and activation of TRPM8 ion channel
Source: Nat Commun. 2020 Jul 29;11:3790. doi: 10.1038/s41467-020-17582-x (PMC7391767; doi:10.1038/s41467-020-17582-x)
Supplement: Supplementary file 4 — Description of Additional Supplementary Files [file 41467_2020_17582_MOESM4_ESM.pdf]

**Description of Additional Supplementary Files**

**File Name**: Supplementary Data 1
**Description:** Menthol docked to the TRPM8 channel in the ligand activated state. The residues from the beginning of S1 to the end of TRP box are included for menthol docking. This is the model shown in Fig. 1e, Fig. 2a and Fig. 3a.
